# **Analysis of disparities in the utilization of virtual prenatal visits in pregnancy**

**DOI:** 10.1016/j.xagr.2022.100142

**Published:** 2022-12-05

**Authors:** Raven Batshon, Rosalyn Maben-Feaster, Carrie Bell, Joanne Motino Bailey, Anca M. Tilea, Michelle H. Moniz, Alex F. Peahl

**Affiliations:** Department of Family Medicine, University of Michigan, Ann Arbor, MI; 2 Department of Obstetrics and Gynecology, University of Michigan, 2800 Plymouth Rd., Ann Arbor, MI 48109

**OBJECTIVE:** The expansion of virtual prenatal care, including telephonic and video follow-ups, has the potential for many notable benefits, including reducing barriers to accessing care such as missed work and travel burden, improving management of chronic diseases and pregnancy complications,^1^ and contributing to high patient and provider satisfaction with prenatal care delivery.^2^^,^^3^ However, virtual care could exacerbate existing healthcare disparities for patients lacking digital access. Published studies on virtual prenatal visits largely describe its use during the COVID-19 pandemic,^4^ but little is known about virtual prenatal visit use outside of an immediate public health crisis.^5^

In March 2020, we launched a hybrid prenatal care plan (in-person and virtual visits) for average-risk pregnant people.^6^ Average risk was defined as ≤1 visit with a maternal-fetal medicine specialist. In March 2021, with relaxation of social distancing measures, we launched an updated model, the Michigan Plan for Appropriate Tailored Healthcare in Pregnancy (MiPATH), which incorporates patients’ preferences for medical care, education, and social support. For medical care, MiPATH includes 5 in-person visits and 3 visits that can be completed virtually or in-person based on shared decision-making between the patients and providers^7^ (Appendix A). Patients with comorbidities or pregnancy complications receive more visits. Virtual visits begin after perceived fetal movement (>20 weeks), so verification of fetal well-being with Doppler is unneeded. A home blood pressure cuff is required, with workflows available to help patients obtain devices. MiPATH has been implemented with >150 providers in 12 clinics. We sought to assess differences in virtual prenatal visit use by key patient demographic characteristics following the immediate public health crisis.

**STUDY DESIGN:** We conducted a single-site, retrospective cohort study of all MiPATH-eligible pregnant patients (ie, those at average risk as defined above) who were >20 weeks gestational age presenting for prenatal care from March 2021 (program launch) to February 2022.

We used electronic health record data to obtain patient characteristics and use data. We compared pregnant people who used the hybrid model (completed ≥1 virtual visit) with those who used in-person care only using basic descriptive statistics and logistic regression models adjusted for all patient characteristics. Analyses were performed using SAS,version 9.4, (SAS, Cary, NC). Statistical significance was set at *P*<.05 with 2-sided tests. The study was exempted by the University of Michigan Institutional Review Board (HUM00205831).

**RESULTS:** Of the 4528 pregnant people seen for prenatal care during the study period, 3880 were MiPATH-eligible; most of them were White (2808/3880, 72.4%), non-Hispanic (3641/3880, 93.8%), and commercially insured (2522/3880, 65.0%), and 1327 (34.2%) completed ≥1 virtual visit (Table). Compared with those who were not eligible, more patients who were MiPATH-eligible were aged 30 to 34 years, partnered, and commercially-insured, whereas fewer patients were Black and multiparous (Appendix B). Of those who were MiPATH-eligible, more commercially-insured, multiparous, and partnered pregnant people completed ≥1 virtual prenatal visit. In adjusted regression models, non-Hispanic (adjusted odds ratio [aOR], 2.35; 95% confidence interval [CI], 1.00–5.50) and multiparous (aOR, 1.30; 95% CI, 1.13–1.50) pregnant people were more likely to complete ≥1 virtual visit, whereas those who were publicly insured were less likely to complete a virtual visit (aOR, 0.81; 95% CI, 0.67–0.99).

**Table:** Patient demographics and adjusted odds ratios for those who completed ≥1 visit vs those with no virtual visits

Group patient characteristics	Total^a^ (N=3880)	No virtual visits^a^ (n=2553)	≥1 virtual visit^a^ (n=1327)	*P* value^b^	Adjusted odds ratio (95% CI)
Age (y)				<.05	
≤20	62 (1.6)	50 (2.0)	12 (0.1)		ref
21–29	1204 (31.0)	816 (32.0)	388 (29.2)		1.74 (0.91-3.33)
30–34	1547 (39.9)	1001 (39.2)	546 (41.1)		1.81 (0.94-3.48)
35–39	909 (23.4)	580 (22.7)	329 (24.8)		1.80 (0.93-3.49)
≥40	158 (4.1)	106 (4.2)	52 (3.9)		1.52 (0.73-3.14)
Race				1.92	
White	2808 (72.4)	1830 (71.7)	978 (73.7)		ref
Black or African American	462 (11.9)	321 (12.6)	141 (10.6)		0.91 (0.73-1.14)
Other^c^	610 (15.7)	402 (15.7)	208 (15.7)		1.01 (0.83-1.25)
Ethnicity				.35	
Hispanic	209 (5.4)	137 (5.4)	72 (5.4)		ref
Non-Hispanic	3641 (93.8)	2400 (94.0)	1241 (93.5)		2.35 (1.00-5.50)
Other^d^	30 (0.8)	16 (0.6)	14 (1.1)		0.89 (0.66-1.20)
Primary language				.289	
English	3703 (95.4)	2430 (95.2)	1273 (95.9)		1.42 (0.95-2.11)
Non-English	177 (4.6)	123 (4.8)	54 (4.1)		ref
Insurance				<.05	
Commercial	2522 (65)	1580 (61.9)	942 (71.0)		ref
Medicaid	741 (19.1)	507 (19.9)	234 (17.6)		0.81 (0.67-0.99)
Uninsured	603 (15.5)	458 (17.9)	145 (10.9)		0.54 (0.44-0.66)
Unknown	14 (0.4)	8 (0.0)	6 (0.5)		1.28 (0.44-3.73)
Parity				<.05	
Nulliparous	2287 (58.9)	1445 (56.6)	842 (63.5)		ref
Multiparous	1593 (41.4)	1108 (43.4)	485 (36.5)		1.30 (1.13-1.50)
Marital status				<.05	
Married or significant other	1664 (42.9)	1060 (41.5)	604 (45.5)		ref
Single or separated or other	1073 (27.7)	718 (28.1)	355 (26.8)		0.97 (0.82-1.16)
Unknown	1143 (29.5)	775 (30.4)	368 (27.7)		0.88 (0.75-1.04)

*CI*, confidence interval; *ref*, referent interval.

a Data are presented as number (percentage)

b *P* value for bivariate analysis calculated using chi-square tests

c Institutional standard selections for race (American Indian or Alaskan Native, Asian, Native Hawaiian or Other Pacific Islander, multiracial, self-described other, unknown, choose not to disclose)

d Institutional standard selections for ethnicity (self-described other, unknown, choose not to disclose).

**CONCLUSION:** Our data demonstrate significant use of hybrid prenatal care, with more than one-third of pregnant people completing ≥1 virtual visit. Despite the promise of virtual visits for alleviating barriers to care and access disparities, we saw lower uptake in key demographic groups, including those with public insurance and Hispanic or Latinx and nulliparous pregnant people. These differences may be driven by patient preference, such as a desire for in-person contact^8^ or disproportionate digital access barriers.^4^ Future research must explore drivers and effects of differential hybrid care use to inform systems of prenatal care delivery that promote patient-centered, equitable access and outcomes.

## Appendix Supplementary materials

Image, application 1

Image, application 2

## Footnotes

Supplementary material associated with this article can be found in the online version at doi:10.1016/j.xagr.2022.100142.
