# Supplementary material for: Analysis of disparities in the utilization of virtual prenatal visits in pregnancy
Source: AJOG Glob Rep. 2022 Dec 5;3(1):100142. doi: 10.1016/j.xagr.2022.100142 (PMC9823200; doi:10.1016/j.xagr.2022.100142)
Supplement: Supplementary file 1 [file mmc1.pdf]

Status Active PolicyStat ID 11968791

MICHIGAN MEDICINE
UNIVERSITY OF MICHIGAN

Origination	12/2013
Effective	06/2022
Last Revised	05/2021
Area	Ambulatory Patient Care
Applicability	UMHS-Clinical Public
References	Guideline

Ambulatory Adult Prenatal Care Guideline

New Prenatal Care Delivery Model-MiPATH

Authors: Mark C. Chames, MD (Lead), Joanne M. Bailey, CNM, PhD, Grant M. Greenberg, MD, MA, MHSA, R. Van Harrison, PhD, Jocelyn H. Schiller, MD, Christa B. Williams, MD (Consultant)

COPY

Patient population: Women of childbearing age, pregnant women, and their fetuses.

A. Promote maternal and infant health.
B. Reduce maternal mortality and morbidity and fetal loss.
C. Reduce preterm birth, intrauterine growth restriction, congenital anomalies, and failure to thrive.

Key Points:
Prenatal care summary. Table 1 summarizes the main aspects of prenatal care from preconception through delivery (history and examination; testing and treatment; education and planning).
Fetal surveillance. Table 2 shows the common indications for antepartum fetal surveillance with nonstress testing and amniotic fluid index (AFI), the gestational ages at which to initiate testing, and the frequency of testing.
Referral. Table 3 summarizes the indications for referral.
Important care aspects:
Assess risk factors. For all women, perform a history and physical that includes a risk assessment with a goal of identifying risk factors for adverse pregnancy outcome *[I-D]*. Review the patient's medical history and any prior pregnancy and delivery records. Clearly document risk factors and add them to the patient's problem list.

- Screen for tuberculosis in pregnant women at high risk for TB.
- Refer hepatitis B carriers to hepatology due to their long term risk for cancer and cirrhosis.
- Screen all patients for depression during the third trimester.

- Provide contraceptive counseling during the third trimester teaching.
- Review future chronic disease risks with patients during pregnancy and at the postpartum visit.

Progesterone therapy. Progesterone should be offered to patients who have a history of prior spontaneous preterm birth or who are found to have a shortened cervix on ultrasound *[I-A]*.

STI testing. Test all women for sexually transmitted infections, including HIV twice during pregnancy. Patients at risk for STIs during pregnancy should be retested again late in the third trimester *[I-A]*.

Estimated date of delivery (EDD). Establish a patient's EDD prior to 20 weeks, with consideration given to menstrual history, mode of conception, and sonographic findings using standardized criteria (see page 14) *[I-C]*.

Tdap vaccination. Offer Tdap vaccination to all women. Administering Tdap at 27-36 weeks facilitates passive pertussis immunization of newborns *[I-D]*, and administering it around 32 weeks may optimize maternal antibody formation peaking at normal time of delivery.

No non-medically-indicated delivery < 39 weeks. Non-medically-indicated planned delivery before 39 weeks' gestation is contraindicated *[III-B]*.

*** Strength of recommendation:**

I = generally should be performed; II = may be reasonable to perform; III = generally should not be performed.

Level of evidence supporting a diagnostic method or an intervention:

A = randomized controlled trials; B = controlled trials, no randomization; C = observational trials; D = opinion of expert panel

COPY

Table 1. Guidelines for Prenatal Care*

Gestational Age	History and Examination	Testing and Treatment	Education and Planning
**Preconception - 12 weeks**	Medical history including menstrual, sexual, immunization, infectious exposure and risk<br>Obstetrical history<br>Family and genetic history<br>Psychosocial history including tobacco, alcohol, drugs, employment, and nutrition<br>Depression screen<br>Intimate partner violence (IPV) screen<br>Current pregnancy symptoms	Blood type and Antibody Screen<br>Hemoglobin / Hematocrit / Platelet count<br>Rubella titer (vaccinate **before** conception †)<br>Hepatitis B Surface Antigen<br>Hepatitis C Antibody<br>HIV<br>STI screening	Counsel on significant positive findings elicited by history, physical, or test results<br>Review test results if available<br>Review dating criteria †<br>Screen for aneuploidy<br>Discuss:<br>-Nutrition in pregnancy (including recommendations for folate and calcium, and cautions regarding mercury in fish and pathogens in uncooked or unpasteurized foods)<br>-Weight gain in pregnancy<br>-Work related exposures or risks

	Complete physical exam including height, weight, BMI, blood pressure, and pelvic examination †	(Gonorrhea, Chlamydia, Syphilis)<br>Urine culture at first prenatal visit<br>Cervical cancer screening †<br>Cystic fibrosis (CF) carrier screening<br>Screening for spinal muscular atrophy (SMA)<br>Hemoglobin electrophoresis †<br>Other genetic screening (Tay-Sachs, etc.) †<br>Diabetes testing †<br>Varicella titer (vaccinate **before** conception) †<br>Tuberculosis testing †<br>Aneuploidy screening†<br>Influenza vaccination †	-Recent or planned travel with regard to Zika and other infectious disease risks or exposures<br>-Breastfeeding<br>-VBAC/TOLAC †<br>Provide obesity counseling †<br>Refer for genetic counseling †<br>Refer to a high-risk provider †
**12-16 weeks**	Current pregnancy symptoms<br>Interim medical, psychosocial, and nutritional evaluation<br>Weight and blood pressure<br>Fetal heart rate	Aneuploidy screen †<br>Diabetes screening at 12 weeks †<br>Influenza vaccination †	Review test results<br>Discuss:<br>-Physical changes<br>-Safe sex and sexuality during pregnancy<br>-Exercise and fitness during pregnancy<br>-Managing work during pregnancy<br>-Seatbelt use in pregnancy
**16-22 weeks**	Current pregnancy symptoms<br>Interim medical, psychosocial, and nutritional evaluation	Ultrasound<br>Progesterone for prevention of recurrent preterm birth †	Review test results<br>Review dating criteria<br>Discuss<br>-Signs of complications, including preterm labor and preeclampsia

COPY

	Weight and blood pressure<br>Fetal assessment including fetal heart rate and growth	Influenza vaccination †	-Childbirth classes<br>-Common discomforts in pregnancy<br>-Emotional changes in pregnancy<br>-Trauma protocol in pregnancy<br>Provide directions to the Birth Center
**22-28 weeks**	Current pregnancy symptoms<br>Interim medical, psychosocial, and nutritional evaluation<br>Weight and blood pressure<br>Fetal assessment including fetal heart rate and growth	Diabetes screening at 24-28 weeks<br>Hemoglobin / Hematocrit / Platelet count at 24-28 weeks<br>HIV<br>Hepatitis B<br>Syphilis<br>Antibody Screen at 24-28 weeks in Rh negative women †<br>Influenza vaccination †	Review test results<br>Discuss:<br>-Signs of complications including preterm labor and preeclampsia<br>-Parenting, infant classes<br>-Breastfeeding classes<br>-Contraception and family planning<br>-Family adjustment<br>-Work plans<br>-Diet and nutrition in pregnancy<br>-VBAC/TOLAC †
**28-34 weeks**	Current pregnancy symptoms<br>Interim medical, psychosocial, and nutritional evaluation<br>Depression screen<br>IPV screen<br>Weight and blood pressure<br>Fetal assessment including fetal heart rate and growth	Tdap vaccination at 27-36 weeks<br>RhoD Immune Globulin (Rhogam) given at 28-29 weeks in Rh negative women †<br>Influenza vaccination †<br>Nonstress testing after 32 weeks †	Review test results<br>Discuss fetal movement monitoring<br>Provide anticipatory guidance regarding labor and delivery<br>Discuss contraception and family planning<br>Identify a newborn care provider<br>Provide car seat information
**34-38 weeks**	Current pregnancy symptoms<br>Interim medical, psychosocial, and nutritional evaluation<br>Weight and blood pressure<br>Fetal assessment including fetal heart rate, growth, and lie (use of ultrasound to document lie is indicated if uncertain by Leopold's maneuvers or sterile vaginal exam)	Anogenital culture for Group B Streptococcus (GBS) at 35-37 weeks (unless already GBS positive in urine during current pregnancy or prior history of a GBS-affected infant)<br>Nonstress testing †<br>STI screening (Gonorrhea, Chlamydia, HIV, Hepatitis B, Syphilis) repeated in high-risk patients †	Review test results<br>Review signs of labor<br>Discuss:<br>-Infant safety after birth<br>-Caring for self and infant after delivery<br>-Parenting issues including return to work, breastmilk collection and storage, childcare

COPY

		Acyclovir for women with HSV †<br>Influenza vaccination †	
**38 weeks - delivery**	Current pregnancy symptoms<br>Interim medical, psychosocial, and nutritional evaluation<br>Weight and blood pressure<br>Fetal assessment including fetal heart rate, growth, and lie	Offer membrane sweeping<br>Delivery by 41-42 weeks (elective delivery prior to 39 weeks is contraindicated)<br>Nonstress testing †<br>Influenza vaccination †	Review test results<br>Review dating criteria<br>Review signs of labor
**Postpartum visit (at 4 to 8 weeks)**	Blood pressure<br>Depression screen<br>IPV screen<br>Breastfeeding<br>Bleeding<br>Laceration healing<br>Pelvic floor recovery	Influenza vaccination †<br>MMR and varicella vaccinations †<br>Cervical cancer screening †<br>Diabetes test †	Discuss:<br>-Contraceptive initiation<br>-Future pregnancy planning†<br>-Future chronic disease risk†<br>-Healthy lifestyle

- The items listed comprise a broad list of general topics to be covered, and may be based on evidence of varying quality, including expert opinion. Some topics may not be relevant for some individuals, while some clinical scenarios may prompt additional evaluation or education that is not listed here. Emphasize items that are most relevant for your patient.

† These items should be performed when indicated by the clinical scenario.

COPY

Table 2. Common Indications for Fetal Surveillance with Nonstress Test and Amniotic Fluid Index

Diagnosis	Gestational Age to Initiate Testing	Frequency of Testing (NST and AFI)
Advanced maternal age (≥ 35 years at delivery)	36 weeks	Once a week
Amniotic fluid volume and amniotic fluid index (AFI)		
Mildly decreased (AFI < 8 cm)	Time of diagnosis	Once a week
Oligohydramnios (AFI ≤ 5 cm)	Time of diagnosis	Per high-risk provider
Cholestasis of Pregnancy		
Bile acids < 40		No testing
Bile acids >=40	32 weeks	Once a week
Diabetes		
Gestational, diet controlled	40 weeks	Once a week
Gestational, requiring medication	32 weeks	Twice a week (AFI once

		a week)
Pregestational	32 weeks	Twice a week (AFI once a week)
Fetal Growth Restriction		
Fetal weight 6th to 10th percentile, normal Doppler studies	Time of diagnosis	Once a week
Fetal weight ≤ 5th percentile or abnormal Doppler studies	Time of diagnosis	Per high-risk provider
Hypertension		
Chronic, not requiring medication	32 weeks	Once a week
Chronic, requiring medication	32 weeks	Twice a week (AFI once a week)
Gestational	Time of diagnosis	Twice a week (AFI once a week)
Preeclampsia	Time of diagnosis	Twice a week (AFI once a week)
Obesity, BMI ≥ 40	36 weeks	Once a week
Post-dates (past 40 weeks) pregnancy	41 weeks	Twice a week
	42 weeks	Every other day
Previous Intrauterine Fetal Demise (IUFD)	Two weeks prior to earliest IUFD	Twice a week (AFI once a week)

COPY

Note: These guidelines may be based on data of variable quality, and in some cases represent expert opinion. This list is not intended to be comprehensive, as numerous other indications for testing are accepted in complicated pregnancies.

Table 3. Selected Indications for Consultation and/or Referral to High-Risk Pregnancy Care

**Medical Complications**<br>Carcinoma<br>Gestational diabetes mellitus requiring medication<br>Pregestational diabetes mellitus (with or without need for medication)<br>Severe chronic medical disease<br>Thrombocytopenia, moderate or severe<br>**Past OB/Gyn History**<br>Previous fetal or neonatal demise with continuing cause<br>Previous major operations to the uterus and cervix, including classical (vertical incision) cesarean delivery, cerclage, resection of uterine septum, fibroid removal or myomectomy (but not including LTCS)<br>Prior preterm birth < 34 weeks<br>Recurrent spontaneous abortion (3 or more)

**Current Pregnancy Complications**
Documented serious fetal anomaly (eg, diaphragmatic hernia)
Hyperemesis unresponsive to outpatient therapy
Alloimmunization
Multiple gestation
Second- or third-trimester fetal demise
Preeclampsia with severe features or eclampsia
Shortened cervix ≤ 20 mm identified on ultrasound
Third trimester bleeding due to placenta previa or placental abruption
Vasa previa

Rationale for Recommendations

When to Deliver Care

Evidence is limited as to what represents an adequate number of prenatal care visits. Studies have shown that some prenatal care is better than no prenatal care, and that a visit during the first trimester is especially important. This guideline presents a chronological sequence of prenatal care that is based on scientific evidence, recommendations of the US Public Health Service, clinical judgment regarding effectiveness of identifying and modifying risk, and the success of medical and psychosocial interventions.

The sequence of prenatal care, including History, Examination, Testing, Treatment, Planning, and Education is summarized in Table 1.

Detailed recommendations and the rationale for care are organized into four major time frames:

- Preconception care
- Prenatal visits
- Delivery planning
- Postpartum assessment

These divisions are followed by additional sections on topics that may be relevant at any time:

- Indications for referral to a high-risk provider
- Cultural sensitivity

Preconception Care

Preconception Visit

A preconception visit is recommended for all women planning to become pregnant in order to minimize risk before pregnancy. Elements of care are summarized in Table 1 and detailed below. When a patient expresses a desire for pregnancy, consider the following:

- **History:** Perform and document maternal medical history and risk assessment.
- **Physical exam**: Perform and document a complete physical examination.

COPY

- **Laboratory tests:**
  - Assess infectious disease risk and immunization status for rubella, tuberculosis, HIV, hepatitis B, varicella, herpes, hepatitis C, and Zika. Vaccinate as indicated (eg, if rubella titer is negative, then provide preconception vaccination and advise that pregnancy should be avoided for 4 weeks).
  - Perform the tests recommended in Table 1. Type and screen, HIV, Hepatitis B, RPR, gonorrhea and chlamydia should be retested in pregnancy.
- **Genetic counseling**: Offer screening to couples for cystic fibrosis carrier status and for spinal muscular atrophy. Provide genetic counseling and testing as indicated based on family history and race/ethnicity probabilities (eg, sickle cell, Tay-Sachs).
- **Health promotion:**
  - Encourage healthy behaviors (eg, weight management, exercise, folate supplementation)
  - Discuss risk factor reduction: smoking, alcohol, substance use, and environmental exposures.
  - Review fetal health risks (eg, optimal blood sugar control in patients with diabetes).
  - Counsel patients that Zika virus infection during pregnancy can cause serious birth defects. Zika infection is mainly caused by a bite from a mosquito infected with Zika, but can also occur through sex with a person infected with Zika. Pregnant women should not travel to areas with documented or likely Zika virus transmission. Couples should wait 3 months after possible Zika exposure before trying for pregnancy.

Overlap of Preconception and Prenatal Care

Given that half of all pregnancies are unplanned, in most cases a preconception visit will not have taken place, so all of the content of the preconception visit must be addressed at the first prenatal visit. This limits the opportunity for primary prevention (eg, some vaccinations will no longer be feasible). The elements of preconception visits are discussed in more detail below in the section on prenatal visits.

COPY

Prenatal Visits

General consideration of the initial prenatal visit and frequency of visits is followed by information on each category of care to be provided during prenatal visits: history, physical examination, laboratory and other tests, and health promotion and education. Within each category, specific aspects of care are listed in the general chronologic sequence in which they are performed, with the timing for specific care activities noted in *italics*.

Initial Prenatal Visit and Visit Frequency

Initial prenatal visit. We recommend that the initial encounter of the pregnancy should consist of an intake visit at *6-8 weeks,* followed by an office visit at *8-12 weeks*.

Frequency of visits. For average risk women visits should occur:

- *Every 4-6 weeks through 34 weeks'* gestation

- *Every 2 weeks through 37 weeks'* gestation
- *Every week after 38 weeks'* gestation

For patients requiring additional surveillance, visit frequency can be tailored individually.
Prenatal care visits should be allotted enough time to facilitate maternal and fetal health assessment as well as offer education and anticipatory guidance; we suggest at least 15 minutes. A group model of prenatal care is also an acceptable alternative to individual appointments.

History

Taking and documenting a thorough history is recommended at the *first pregnancy visit* if a preconception visit has not taken place. Review the patient's medical history and any prior pregnancy and delivery records. Clearly document all risk factors in the visit note and add them to the patient's problem list. Key elements of the history are identified in Table 1, including:

Tobacco use/avoidance. Screening for tobacco use is recommended at the *initial visit*. Tobacco use during pregnancy has well known risks including miscarriage, placental abruption, fetal growth restriction, preterm delivery, and sudden infant death syndrome. Cessation of tobacco use is highly recommended. The UMHS clinical guideline "Tobacco Treatment" provides information on assisting patients to quit tobacco use. Non-pharmacological measures are addressed. Nicotine gum, lozenges, and patches can be considered; while use of these during pregnancy has been associated with low birth weight, the risk of tobacco use itself is still greater. Electronic cigarettes should not be recommended or endorsed for smoking cessation.

COPY

Alcohol and substance use/abuse. Alcohol is a known teratogen, and use of alcohol in pregnancy incurs a risk for fetal alcohol syndrome. Similarly, use of narcotics and other controlled or illicit substances can adversely affect fetal well-being. Screen all patients for alcohol and substance use at the *initial visit* by asking the Four P's (Table 4). These are easy to employ and are potentially effective in detecting problems with substance or alcohol use.

Table 4. Alcohol and Drug Use Screening Questions

The Four P's. Yes on ≥ 1 indicates potential risk.<br>1. Have you ever used drugs or alcohol during this **P**regnancy?<br>2. Have you had a problem with drugs or alcohol in the **P**ast?<br>3. Does your **P**artner have a problem with drugs or alcohol?<br>4. Do you consider one of your **P**arents to be an addict or alcoholic?

Patients who screen positive using a standardized methodology should be further evaluated. Consider referral to a trained alcohol or substance abuse counselor or program as well as consultation with a high-risk provider.

Depression. Depression is common in women of childbearing age and during pregnancy. Identifying and treating depression can benefit both the mother in terms of social function during pregnancy and the fetus by decreasing risk for preterm birth and low birth weight.
Women should be screened for depression at the *preconception visit, initial prenatal visit, in the third trimester, and in the postpartum period* using a validated depression scale such as the Edinburgh Postnatal Depression Screen (EPDS) or PHQ-9.

Treatment of depression in pregnancy can include counseling and behavioral techniques, as well as pharmacologic management. Multidisciplinary care is recommended when available. As with any condition during pregnancy, a careful assessment of the risks of medication versus the potential benefits based on the individual situation is required prior to considering pharmacologic treatment.

Intimate partner violence (IPV). IPV occurs with a high prevalence during pregnancy; up to 20% has been reported in some studies. Therefore, screening for IPV is recommended at the *preconception visit, initial prenatal visit, in the third trimester, and in the postpartum period*. Screening at multiple visits can result in a higher rate of detection than does screening only at the initial visit. Screen for intimate partner violence using the questions in Table 5.

Table 5. Screening for Intimate Partner Violence

1. Do you feel you are not treated well by your partner/spouse/family member?
2. Have you been hit, slapped, kicked, pushed, shoved, or otherwise physically hurt by your partner/spouse/family member?
3. Has anyone ever forced you to have sexual activities?

COPY

If any of the Table 5 questions are positive, it is essential to evaluate the safety of the patient and family members. Consider a referral to either adult or child protective services and a referral to social work.

Recurrent preterm birth. Patients with a prior history of spontaneous preterm birth between 20-37 weeks resulting from spontaneous preterm labor or premature rupture of membranes are at risk for recurrent preterm birth. Data regarding the efficacy of prophylactic progesterone is mixed. This should be discussed with the patient and they should be offered progesterone supplementation and undergo serial cervical length assessments from *16 to 24 weeks*.

Patients with a history of prior spontaneous preterm birth prior to 34 weeks should be referred for consultation to a physician trained in the care of high-risk obstetrical patients.

Treatment should begin at *16-20 weeks* with 17 alpha-hydroxyprogesterone caproate (17P) 250 mg intramuscularly once weekly (recommended therapy) or progesterone 100 mg vaginally once daily (alternative therapy). Treatment should be continued until 36 weeks' gestation. If treatment is stopped earlier, the risk of preterm birth may increase.

Herpes simplex virus (HSV). In pregnant women who are known to have genital HSV, the use of prophylactic antiviral medication beginning at *36 weeks' gestation* has been shown to reduce the rate of cesarean delivery, although it has no benefit in terms of neonatal outcome. Recommended therapy is

acyclovir 400 mg by mouth three times daily. Valacyclovir 500 mg by mouth twice daily offers more convenient dosing, but at a greater cost.

Serologic screening for HSV infection in asymptomatic women is not recommended. In symptomatic women, clinical history may be adequate to make a presumptive diagnosis, although confirmation with HSV culture may be considered.

If active herpes lesions are present at time of labor admission, cesarean delivery is preferred as the mode of delivery due to uncertainty of the risk from primary versus secondary HSV infection. The risk for neonatal HSV infection, however, is low even if lesions are noted incidentally after a vaginal delivery. The newborn provider should be made aware if delivery occurs in the setting of a recent or active genital HSV outbreak.

Physical Examination

Mother. The mother's examination should include:

- **Height** measured at the *initial visit*.
- **Weight** recorded at *each visit*. Evaluating weight gain is a simple and appropriate measure to potentially reduce risk for complications. If a patient experiences excessive or poor weight gain, then additional nutritional guidance may be necessary. A nutrition consultation can be considered.
- **Body mass index (BMI)** calculated at the *initial prenatal visit*.
- **Blood pressure** measured at *each visit*.
- **A physical exam** performed at the *initial visit*. If breast and pelvic examinations have been recently performed, the clinician may consider omitting these portions of the exam.

COPY

Fetus. The examination of the fetus should include:

- **Fetal heart rate** assessed *at each visit*. The normal fetal heart rate varies by gestational age, but typically is between 120 and 160 beats per minute. If no fetal heart rate is detected using Doppler or fetoscope by 12 weeks, then an ultrasound should be performed for fetal heart rate assessment.
- **Fundal height or assessment of fetal growth** recorded at *each visit from 20-36 weeks*. If fundal height differs by 4 cm or more from the corresponding gestational age, then an ultrasound should be ordered to assess fetal growth and amniotic fluid volume. If an ultrasound has recently been done to assess fetal growth, measurement of fundal height provides no additional benefit. If measuring fundal height is not possible, or in cases where BMI ≥ 40, an ultrasound at 28 weeks and 34 weeks for growth assessment is suggested.
- **Fetal presentation** assessed *after 34 weeks* by Leopold's maneuvers and/or ultrasound. If a fetus remains breech at 37 weeks, an external cephalic version should be offered to appropriate candidates. If version is unsuccessful, a scheduled term (≥ 39 weeks) cesarean delivery is recommended.

Obesity. Obese pregnant women (BMI ≥ 30 kg/m^2) require additional considerations in providing care.

- **Counsel** at *the initial visit* regarding:

- Weight gain, nutrition, and regular exercise.
- Associations of obesity with fetal malformation, fetal macrosomia, maternal hypertensive disorders, gestational diabetes, obstructive sleep apnea (OSA), increased cesarean delivery rate, and intrapartum and operative complications.

- **Test for diabetes** at the *initial visit*.
- **For BMI:**
  - ≥ 40 kg/m^2: sonographic evaluation of fetal growth is suggested *at 28 and 34 weeks*.
  - ≥ 50 kg/m^2: sonographic evaluation *at 28 and 34 weeks* is suggested as above, plus an evaluation by anesthesiology *in the third trimester*.
  - At any BMI, if the body fat distribution limits clinical assessment of fetal growth, serial sonographic evaluation of fetal growth is suggested.
- **If OSA is suspected, or if known OSA is not treated**, consider consultation with a sleep medicine expert.

Hypertensive disorders of pregnancy. Hypertension in pregnancy is defined as a systolic pressure greater than 140 mm Hg and/or a diastolic pressure greater than 90 mm Hg recorded on at least two separate occasions at least four hours apart. Hypertensive disorders noted during pregnancy are divided into 4 categories: preeclampsia-eclampsia, chronic hypertension, chronic hypertension with superimposed preeclampsia, and gestational hypertension.

- **Preeclampsia** is hypertension identified after 20 weeks' gestation in the presence of new onset proteinuria (protein/creatinine ratio on a random specimen with a cut-off of 0.3, or excretion of 300 mg/24 hours on a timed urine), thrombocytopenia (< 100,000/mcL), impaired liver function (elevation of transaminases to twice the normal concentration), new-onset renal insufficiency (creatinine > 1.1 mg/dL or doubling of serum creatinine in the absence of other renal disease), pulmonary edema, or new onset cerebral or visual disturbances.
- **Chronic hypertension** is hypertension that predates pregnancy. In patients without documentation of blood pressure prior to conception the diagnosis is suspected in the presence of two elevated pressures prior to the 20th week of gestation in the absence of multiple gestation or gestational trophoblastic disease.
- **Chronic hypertension with superimposed preeclampsia** is chronic hypertension in association with preeclampsia.
- **Gestational hypertension** is hypertension identified after 20 weeks' gestation in the absence of proteinuria or other systemic findings suggestive of preeclampsia.

If a patient's blood pressure is elevated, evaluation for proteinuria is recommended. A quantitative test, such as a protein/creatinine ratio on a random specimen or a timed urine collection, is preferred. Significant proteinuria may also be documented at a level of ≥ 1+ protein on dipstick, but due to variability in this test, confirmation is suggested using one of the previously mentioned techniques. If the protein/creatinine ratio is normal and clinical suspicion is high, consider repeating the test or performing a timed urine collection.

Additional laboratory tests that may aid in the evaluation of suspected preeclampsia include a complete

COPY

blood count with platelets, AST, ALT, and serum creatinine. Serum uric acid has a low positive predictive value and is generally not helpful.
The complete management of hypertension in pregnancy is beyond the scope of this guideline. For detailed recommendations regarding the care of these patients, the use of another resource is recommended. The following principles may aid in the care of these patients.

Patients at high risk for development of preeclampsia include patients with a history of prior preeclampsia, diabetes, chronic hypertension, renal disease, autoimmune disorder, or multiple gestation. Treatment with low-dose aspirin (81 -162mg by mouth once daily) beginning at 12 weeks' *gestation is recommended to reduce the* risk for development of preeclampsia in these patients. Routine aspirin prophylaxis has not been shown to be beneficial in low-risk patients.

Gestational hypertension and preeclampsia without severe features are indications for sonographic evaluation of fetal growth and antepartum surveillance (Table 2). Patients should be followed with serial blood pressure assessment. Patients with gestational hypertension or preeclampsia should undergo *weekly laboratory evaluation*. Delivery is indicated *at ≥ 37 weeks*.

Preeclampsia with severe features is preeclampsia characterized by systolic blood pressure ≥ 160 mm Hg, diastolic blood pressure ≥ 110 mm Hg, thrombocytopenia, severe persistent right upper quadrant or epigastric pain, impaired liver function, new-onset renal insufficiency, pulmonary edema, or new onset cerebral or visual disturbances. If this develops *at any gestational age*, admit the patient to the hospital for further evaluation and management. Bed rest does not alter the course of preeclampsia.

Laboratory and Other Tests

Most initial prenatal laboratory tests should be collected during the prenatal period, even if previously collected during a preconception visit.

Blood type and antibody status. Maternal ABO and Rh blood type and blood antibody status should be documented at the *first prenatal visit*.

Women who are Rh negative and not Rh sensitized should have a repeat antibody screen performed *at 24-28 weeks* then receive RhoD Immune Globulin (Rhogam) 300 mcg intramuscularly *at 28 to 29 weeks* prenatally. If the newborn is Rh positive, RhoD Immune Globulin (Rhogam) should be administered again *within 72 hours of delivery* at a dose dictated by blood bank studies.

Unsensitized patients who are Rh negative and experiencing vaginal bleeding during pregnancy should receive RhoD Immune Globulin (Rhogam) *within 72 hours* of any episode of vaginal bleeding due to concern for feto-maternal hemorrhage. If RhoD Immune Globulin (Rhogam) is given during the prenatal period for this indication, it need not be routinely readministered until the patient is beyond *28 weeks' gestation* and *12 weeks have passed* since administration. If concern exists for ongoing hemorrhage, it is reasonable to check the maternal indirect Coombs test *every 3 to 4 weeks*. If the test is positive, RhoD Immune Globulin (Rhogam) is still present and repeat dosing is not necessary. If the test is negative, repeat dosing of RhoD Immune Globulin (Rhogam) is suggested.

Hemoglobin and hematocrit. Hemoglobin and hematocrit should be checked at the *first prenatal visit*

COPY

and *repeated once after 24 weeks*,
If the hematocrit is less than 33, supplemental iron is recommended. Newer studies suggest that oral iron taken every other day is better tolerated and leads to similar improvement in hemoglobin as daily or twice daily dosing. For hematocrit <30, repeat a hemoglobin measurement and ferritin levels *2-4 weeks* after beginning supplementation to assure an appropriate response. If hemoglobin/hematocrit has not improved by 1 gram/3%, then evaluate how the iron is being taken.

Patients receiving iron supplementation should be counseled on side effects of treatment, including constipation, as well as treatments for this. They should also be counseled that consuming vitamin C in the form of citrus or a vitamin C supplement at the time of iron dosing improves iron absorption. In contrast, taking iron within *2 hours* of consuming calcium supplements, dairy, soy, spinach, coffee, or tea can impair the absorption of iron.

For patients who have a hematocrit < 27% that is either persistent or diagnosed *after 30 weeks' gestation*, intravenous iron therapy should be encouraged.

Platelet count. Platelet count should be performed at the *initial visit*. Thrombocytopenia is classified as mild (100,000-149,000/mcL), moderate (50,000-99,000/mcL) or severe (< 50,000/mcL). Patients with new-onset mild thrombocytopenia at the initial evaluation may have idiopathic thrombocytopenic purpura (ITP) and should have repeat evaluation *at 28 weeks*. If at any time the platelet count is in the moderate to severe range, consider consultation with a high-risk obstetric provider and/or hematology.

Gestational thrombocytopenia is the most common cause of thrombocytopenia in pregnancy, usually presenting in the third trimester. Platelet levels do not typically fall below 70,000/mcL. Gestational thrombocytopenia is likely in patients who develop mild thrombocytopenia in the third trimester, are asymptomatic, and have no history of bleeding or thrombocytopenia prior to pregnancy other than during a previous pregnancy or during the *first 12 weeks postpartum*. These patients require no special evaluation or treatment.

Rubella titer. A rubella titer should be performed for all women at the *initial visit*. Non-immune women should be vaccinated at least *28 days prior to conception* or should avoid exposure and be vaccinated in the immediate postpartum period. If rubella immune status is documented within the year prior to conception, then testing need not be repeated prenatally.

Screening for sexually transmitted infection (STI). STI screening should be performed on all women at the *first prenatal visit*. Repeat screening for HIV, Hepatits B, and syphilis is recommended at 24-28 weeks. If testing is positive at any time, treatment, counseling, and referral of partner(s) for testing and treatment are recommended.

In high-risk patients, repeat screening for all sexually transmitted infections should be completed *at 32-36 weeks' gestation*.

Neisseria gonorrhoeae and Chlamydia trachomatis. Women with a positive test should be treated and followed with a test of cure *at least four weeks after* treatment due to risk for complications resulting from persistent or recurrent infections. Infected pregnant women should abstain from intercourse pending test of cure. To prevent reinfection, sexual partners should also be treated, with consideration given to expedited partner therapy.

COPY

Syphilis. Syphilis screening using a serologic test (ie, rapid plasma reagin [RPR]) should be performed on all patients at the beginning of pregnancy and repeated at 24-28weeks. This should be *repeated again at 32-36 weeks in women who are high-risk* for infection. Positive (reactive) tests should be confirmed by a treponemal test (ie fluorescent treponemal antibody [FTA]) before treatment. Women with confirmed positive serology should be treated with penicillin. Follow-up serologic tests should be obtained after treatment to document decline in titers. See the CDC Sexually Transmitted Diseases Treatment Guidelines for details.

Routine screening of asymptomatic pregnant patients for trichomoniasis or mycoplasma infections is not recommended. If trichomoniasis is diagnosed, treatment is recommended only if the patient is symptomatic.

Hepatitis B surface antigen (HBsAg). Testing for HBsAg should be performed at the *first prenatal visit* and repeated 24-28weeks pregnancy on all women regardless of history of hepatitis B immunization, and carrier status should be documented in the delivery record. In patients at high risk, testing should be *repeated again at 32-36.* Patients who test positive for HBsAg should undergo testing for hepatitis B e-antigen (HBeAg), HBV DNA quantification, and ALT. These women should be referred to hepatology at diagnosis due to their long-term risk of cirrhosis and cancer. Their infants should receive both hepatitis B immune globulin and hepatitis B vaccine *within 12 hours of birth.*

Hepatitis C. Presence of hepatitis C antibody should be assessed for all pregnant women. Women at high risk for Hepatitis C infection should be tested again *at 32-36 weeks*. Women at high risk include those with a history of intravenous drug use or a hepatitis C infected partner.

Vertical transmission (from mother to infant) of hepatitis C virus (HCV) occurs exclusively among women with detectable HCV RNA in the blood. Patients who have detectable HCV RNA, or a suspicion of recent or acute HCV, should be referred to hepatology for care. Hepatitis C is not a contraindication to vaginal delivery or to breastfeeding.

HIV. The baseline rate of perinatal transmission of HIV from an infected woman to her infant is 25-30%. Maternal anti-retroviral therapy has been shown to reduce this rate. HIV testing is recommended for all women at the *initial visit*. Offer testing again in the *third trimester* and at the *onset of labor* if initial testing was declined.

HIV testing should be for all patients at 24-28weeks and *repeated again at 32-36 weeks in women who are high-risk* for infection. This includes patients with a history of any STI, intravenous drug use, more than one sex partner in the last six months, recent blood transfusion, or an HIV-infected partner.

Urine culture. Screen women for asymptomatic bacteriuria with urine culture at the *first prenatal visit*. Evidence is insufficient to recommend for or against repeat screening throughout the remainder of the pregnancy.

Asymptomatic pregnant women with urinary bacterial colony counts < 100,000 CFU/mL should not be treated with antibiotics, as no benefit is seen in prevention of adverse maternal or fetal outcomes. This recommendation applies to all bacterial isolates, including Group B Streptococcus, although the presence of GBS at any level in the urine should be documented, as this will necessitate intrapartum antibiotics.

COPY

If bacterial colony counts are > 100,000 CFU/mL, treatment at the time of diagnosis is recommended. Evidence is insufficient to recommend a test of cure after completion of antibiotic therapy, except in the case of GBS bacteriuria, for which a test of cure is recommended.

Cervical cancer screening. Women who are current with routine screening for cervical cancer do not need to undergo additional testing. Rates of false positive cervical cytology increase in pregnancy; however, pregnancy presents an opportunity to detect disease in women not previously screened.

Genetic screening. The family history, including ethnic background, of all patients should be determined at the *initial visit*. Patients at risk for carrying a genetic disorder should be offered testing for that disorder. Abnormal results suggesting that the patient is a carrier should prompt partner evaluation. If both partners are carriers, consider referral for genetic counseling.

Offering screening for genetic carrier status is recommended for the conditions listed below. Performance of a larger panel that screens for more diseases may be more cost effective than ordering these tests individually. Ideally, this testing should be performed prior to pregnancy. Insurance coverage for these assays is variable.

Cystic fibrosis (CF). All couples should be provided with information about CF and offered carrier screening. Inform patients about the limitations of such testing and that the newborn screen includes testing for the most common gene mutations that can cause cystic fibrosis.

Spinal Muscular Atrophy (SMA). All patients who are pregnant or considering pregnancy should be offered genetic screening for SMA. Note, many insurances are not covering this screen.

Hemoglobinopathies. All women should be screened for hemoglobinopathies with a complete blood count with red blood cell indices along with an assessment of risk due to ethnic/racial background. Hemoglobin electrophoresis should be offered to patients with depressed MCV or MCH, and to all women of African, Mediterranean, Middle Eastern, East Indian, South American, Caribbean, and Southeast Asian descent.

Ashkenazi Jewish descent. Individuals descended from the Ashkenazi Jews of central and eastern Europe (or with partners of such descent) are at risk for several heritable disorders. These individuals should be offered carrier screening for cystic fibrosis as well as Tay-Sachs disease, Canavan disease, and familial dysautonomia. If patients request screening, consider ordering an Ashkenazi genetic panel that screens for Tay-Sachs disease, Canavan disease, and familial dysautonomia as well as multiple other genetic mutations such as mucolipidosis, Niemann-Pick disease type A, Fanconi anemia group C, Bloom Syndrome and Gaucher disease. The panel is more cost-effective than testing for individual disorders.

Diabetes testing. Testing for diabetes is recommended at the *initial prenatal visit* in women with signs or symptoms suggestive of undiagnosed diabetes, as well as those with a history of gestational diabetes (GDM), a first degree relative with diabetes, or other risk factors (eg, age ≥ 35, BMI ≥ 30, inactive lifestyle, prior macrosomic infant, prediabetes, or PCOS). A reasonable approach is to evaluate Fasting Plasma Glucose (FPG) and A1c. The results are interpreted as:

- FPG ≥ 126 mg/dL or A1c ≥ 6.5 is diagnostic of overt diabetes.

COPY

- FPG ≥ 95 mg/dL, but < 126 is diagnostic of GDM.

Patients who have risk factors noted above, but have normal FPG (< 95 mg/dL) and A1c of 5.7-6.4 should be counseled on dietary and lifestyle modification and should undergo an oral glucose challenge test (GCT) at *12 weeks' gestation*, using the procedures described below in the section on "Screening for gestational diabetes." If GDM is not diagnosed, a second GCT should be *repeated at 24-28 weeks* or any time a patient has signs or symptoms suggestive of hyperglycemia.

In patients for whom testing of both the FPG and A1c is not logistically plausible, an A1c alone may be performed, although sensitivity of the protocol may be diminished.

Preconception evaluation: When evaluating patients *before conception*, the same protocol may be performed using the following interpretation:

- Pre-diabetes is diagnosed if FPG is 100-125 mg/dL or A1c is 5.7-6.4%
- Diabetes is diagnosed if FPG ≥ 126 or A1c ≥ 6.5%

Varicella. Prenatal assessment of varicella immunity is recommended at the *initial visit*. The following are considered evidence of immunity.

- History of varicella vaccine administration
- History of varicella disease or herpes zoster
- Laboratory evidence of immunity or confirmation of disease

COPY

Varicella tends to occur at an older age in tropical areas, as compared to temperate areas where varicella tends to be disease of childhood. Recent immigrants from tropical areas are therefore less likely to have contracted varicella in childhood and are more likely to be non-immune if unvaccinated.

Varicella non-immune women should be vaccinated, receiving the last dose at least 1 month *prior to conception,* or they should avoid exposure and receive the first dose of vaccine in the *immediate postpartum period.*

Tuberculosis. All women at high risk should be screened for tuberculosis at the *initial visit* using either a Mantoux intradermal test with purified protein derivative (PPD), or an interferon-gamma release assay (IGRA) such as the Quantiferon-Gold test. High-risk groups for tuberculosis include individuals who:

- Live in close contact with individuals known or suspected to have tuberculosis
- Have medical risk factors known to increase risk of disease if infected (eg, immunocompromised state, HIV)
- Were born in a country with high tuberculosis prevalence
- Are alcoholics
- Are intravenous drug users
- Are residents of long term care facilities, including correctional facilities
- Are healthcare professionals working in high-risk healthcare facilities, if not recently screened.

Screening for aneuploidy and neural tube defects. All pregnant women presenting for care, regardless of age, should be offered aneuploidy screening options. All women considered at high risk due to

maternal age (age 35 or older at delivery) or personal or family history should be offered consultation with a genetic counselor. Prior to performing a screening test, discuss with the patient the possible results and subsequent evaluation. Screening options that should be offered include:

- **No testing**
- **Non-invasive prenatal testing (NIPT)**: This approach evaluates cell-free placental DNA in maternal plasma after 10 weeks' gestation as a screen for Down syndrome, Trisomy 13, Trisomy 18, and sex chromosomal abnormalities. As a screen for Down syndrome the sensitivity exceeds 99%, with a false positive rate of approximately 1/1000.
- **First trimester screen (FTS)**: This screening test that can identify about 85% of pregnancies with Down syndrome and 97% with trisomy 18, with a false positive rate of 5%. This test measures blood levels of free beta-hCG and PAPP-A (pregnant associated plasma protein A) at 9-14 weeks. In addition, an ultrasound is performed at 11-14 weeks to assess the nuchal translucency.
- **Quad screen**: This screening test can identify about 80% of fetuses with Down syndrome, 80% of those with open neural tube defects, and 60% with trisomy 18, with a false positive rate of 5%. It measures blood levels of alpha-fetoprotein, beta-hCG, estriol, and inhibin A. It is performed between 15 and 21 weeks.

If a screening test is positive, then genetic counseling and ultrasound should be offered.

Patients who are at increased risk on the basis of history or the result of one of the above screening tests should be offered genetic counseling. Additional tests that may be made available at the time of genetic counseling include chorionic villus sampling (CVS) and amniocentesis. These are diagnostic procedures that carry risk for pregnancy loss. CVS is performed at 10-13 weeks and amniocentesis is performed after 15 weeks.

COPY

Patients without risk factors who request CVS or amniocentesis should meet with a genetic counselor to review the risks in detail.

Women who undergo NIPT, FTS, or CVS should be offered ultrasound screening for neural tube defects.

Fetal ultrasound. The routine use of screening fetal ultrasound in low-risk women has not been demonstrated to improve long-term outcome. Nonetheless, offering ultrasound imaging is reasonable, given its benefit in terms of dating and identification of anomalies. Ultrasound imaging can confirm pregnancy viability in cases where fetal heart tones are not readily found through use of a Doppler. Ultrasound imaging lowers the rate of induction for presumed postterm (beyond 42 weeks) pregnancy. In women at increased risk for fetal abnormality where an intervention might improve the outcome, an ultrasound should be recommended.

It is reasonable to perform ultrasound at the *initial prenatal visit* in order to evaluate pregnancy viability, dating, and to evaluate for multiple gestation.

A screening fetal anatomic survey is reasonable at *18-20 weeks*.

Cervical ultrasound. Asymptomatic low-risk women who are found to have a shortened cervix in the second trimester are at increased risk for preterm birth. Treatment with vaginal progesterone has been shown to improve pregnancy outcome. We recommend that the cervical length be evaluated at the time

of the screening ultrasound (*18-20 weeks*). Patients found to have a shortened cervix ≤ 20 mm should be offered progesterone therapy. This may be given as micronized progesterone 200 mg intravaginally at bedtime or as progesterone 8% gel (90 mg) intravaginally every morning, continuing treatment *until 36 weeks*. These patients should also be referred to a physician trained in the care of high-risk obstetrical patients.

Screening and care for gestational diabetes. Gestational diabetes mellitus (GDM) is defined as glucose intolerance that begins or is first recognized during pregnancy.

Screening for gestational diabetes. All pregnant women not known to have diabetes should undergo a 50 g oral glucose challenge test (GCT) at *24-28 weeks*, with testing of the plasma glucose at 1 hour. Validated options for the 50 g glucose load include a standardized glucose beverage, 28 Brachs ® jelly beans, or 10 Twizzler ® strawberry twists. A 1-hour glucose result of ≥ 135 mg/dL is considered abnormal and should be followed by a 100 g, 3-hour oral glucose tolerance test (OGTT). If the OGTT finds an elevated fasting plasma glucose (≥ 95 mg/dL) or 2 or more abnormal values after glucose loading (1 hour ≥ 180 mg/dL, 2 hour ≥ 155 mg/dL, 3 hour ≥ 140 mg/dL), then the patient has GDM. Patients with a 1-hour glucose result on the 50 g glucose challenge of ≥ 200 mg/dL are also considered to have GDM and do not need to do the 3-hour OGTT.

Care for patients with GDM. GDM has two phases of risk and treatment:

- Prenatal: GDM is associated with maternal, fetal, and neonatal risks, including polyhydramnios, preeclampsia, cesarean delivery, macrosomia, shoulder dystocia, birth injury, hyperbilirubinemia, hypoglycemia, respiratory distress syndrome, and childhood obesity. Review these risks and refer for dietary counseling and instruction in home blood glucose monitoring. If the patient cannot maintain a fasting blood sugar < 95 mg/dL or a 2-hour postprandial glucose < 120 mg/dL, then pharmacologic therapy should be considered.
- Postpartum. Patients with a history of GDM are at high risk for developing overt diabetes. Perform a 2-hour (75 g) glucose tolerance test at *6-weeks postpartum*. Counsel all patients regarding long-term risks. Recommend a minimum of 150 minutes of exercise per week, spread over 3-5 days.

Antepartum fetal surveillance. Antepartum surveillance should be initiated for pregnancies at increased risk for uteroplacental insufficiency. Table 2 shows common indications for surveillance with nonstress testing and amniotic fluid index, the corresponding gestational ages at which to initiate testing, and the recommended frequency of testing.

Anogenital culture for Group B streptococcus (GBS). Screen all pregnant women for GBS, except for those who have already had isolation of GBS in the urine during the current pregnancy and those with a previous infant affected by invasive GBS disease. Obtain anogenital GBS cultures with antibiotic sensitivity testing at *35-37 weeks' gestation*. A positive culture indicates the need for intrapartum antibiotic treatment. If the culture is negative and the patient has not delivered within 5 weeks of the initial sample, obtain another sample.

The following patients should always be treated with intrapartum antibiotics:

- Prior newborn child with invasive neonatal GBS disease

COPY

- Preterm labor *less than 37 weeks* in the absence of a negative GBS screen unless delivered via cesarean delivery with intact membranes and no labor
- Ruptured membranes ≥ *18 hours* at any gestational age when GBS screening culture status is unknown or unavailable.
- Fever in labor > 38 degrees Celsius (100.4 degrees Fahrenheit) when GBS screening culture status is unknown or unavailable.
- GBS bacteriuria during the current pregnancy.

Tests not recommended. Routine screening for the following is not recommended:

- Vitamin D
- Thyroid stimulating hormone (TSH)
- Toxoplasmosis
- Cytomegalovirus (CMV)
- Herpes simplex virus (HSV)
- Routine urinalysis at prenatal visits.

Health Promotion and Education

COPY

Vaccinations. Recommendations for preconception, antepartum, or postpartum vaccinations include the following.

- Rubella. Non-immune women should receive MMR vaccine *at least 1 month* prior to conception, or they should avoid exposure and be vaccinated in the immediate postpartum period. Non-immune postpartum women should receive a dose of MMR vaccine before discharge from the health-care facility.
- Varicella. Non-immune women should be vaccinated with two doses of varicella vaccine before conception, receiving the last dose *at least 1 month* prior to conception, or they should avoid exposure and be vaccinated in the immediate postpartum period. Non-immune postpartum women should receive the first dose of vaccine before discharge from the health-care facility. The second dose should be administered 4-8 weeks later.
- Influenza. Influenza vaccination is recommended for all women who will be pregnant during influenza season, and may be administered *at any gestational age*. In some studies, maternal influenza vaccination has shown benefits for the child, including a decreased incidence of fetal demise and a decreased likelihood of infant hospitalization for influenza within the first 6 months of life.
- Pertussis. Administer a dose of Tdap during each pregnancy irrespective of the patient's prior history of immunization. To maximize the maternal antibody response and passive antibody transfer to the infant, the CDC recommends immunization *between 27 and 36 weeks,* although it may be administered at any gestational age. For maternal antibody formation to peak around the normal time of delivery, the optimal time for administration may be around 32 weeks.

Nutrition counseling. In addition to general good nutritional counseling, the following are important:

- Folate or folic acid. Folate supplementation before and during pregnancy has been shown to reduce the risk for neural tube defects and is recommended for all patients. While national

guidelines suggest a dose of 0.4 mg daily, evidence suggests that higher doses may confer additional benefit. Folate supplementation at a dose of 1 mg daily is recommended, beginning at least three months prior to conception and continuing through the first trimester. Women with a prior pregnancy complicated by a neural tube defect should supplement their diets with folate 4 mg daily, beginning *at least one month* prior to conception and continuing through the first trimester.

- Calcium supplementation. Calcium supplementation is recommended for women who have a low intake of calcium rich foods. Recommended supplementation: 2 g of elemental calcium daily.
- Multivitamin. The routine use of prenatal multivitamins is not recommended as they have not been shown to improve pregnancy outcome, although they offer a convenient source of folic acid, with most formulations containing 0.8 – 1.0 mg of folate.
- Food with specific risks. Fish provides an excellent source of omega-3 oils, but should be consumed in moderation, avoiding fish high in mercury and other contaminants. See www.michigan.gov/eatsafefish for a current list.
- Certain raw or unpasteurized foods carry an increased risk of causing foodborne illness and should be avoided in pregnancy. See www.foodsafety.gov for current recommendations.

Weight gain in pregnancy. Excessive weight gain during pregnancy increases the risk for complications of delivery from fetal macrosomia, such as labor dystocia, shoulder dystocia, and need for operative delivery. It also increases the risks of maternal gestational diabetes and postpartum obesity. Inadequate weight gain is associated with preterm delivery, intrauterine growth restriction, and low birth weight.

Established parameters for weight gain are based on pre-pregnancy body mass index (BMI). ACOG, IOM, and AAP recommend the following:
Pre-pregnancy BMI (kg/m^2)Recommended Weight Gain
< 19.812.7-18.2 kg (28-40 lbs)
19.8-2611.3-15.9 kg (25-35 lbs)
26-296.8-11.3 kg (15-25 lbs)
> 295.0-9.1 kg (11-20 lbs)

Behavioral counseling and dietary education have been shown to be beneficial for women with BMI < 20 and ≥ 30.

Breastfeeding. Begin breastfeeding education for all pregnant women during the *initial visit* with the clinician. Continuing education throughout pregnancy should be offered to pregnant women who express a desire to breastfeed and for those who are still undecided on feeding method. Breastfeeding provides substantial health benefits for children (decreased ear, respiratory and gastrointestinal infections) and their mothers (decreased ovarian and breast cancer). Feeding infants artificial milk (formula) is associated with an increased likelihood of chronic disease in children (obesity, asthma and diabetes).

Exercise. Exercise during pregnancy is safe and beneficial to both mother and fetus. There is no evidence of risk to fetal well-being or that prolonged activity incurs a higher risk for either pre-term labor or pre-term delivery. Regular (3 or more times weekly) mild to moderate exercise is recommended for all

COPY

healthy pregnant women. The choice and amount of exercise can be tailored to the patient based on their pre-pregnancy activities. Avoid activities that confer inherent risk for abdominal trauma. Avoiding activities at high altitudes (> 10,000 feet [3,000 meters]) is suggested for patients not acclimated to this environment.

Fetal movement counts. Fetal movement is a marker for fetal well-being, so counseling women to assess fetal movement can be potentially beneficial. No specific number of movements should occur within a set time frame; however, a threshold of fewer than 10 movements in 2 hours is a reasonable parameter for further assessment. Patients who experience decreased fetal movement *after 24 weeks' gestation* should contact their clinician immediately for phone triage and possible in-person evaluation. All patients *after 32 weeks' gestation* should be evaluated for fetal well-being in person promptly after reporting decreased fetal movement.

Contraceptive counseling. Discuss postpartum contraceptive options during prenatal care *by 32-34 weeks*. Clinician-initiated discussion is recommended, as patients may not raise the topic. Reviewing options during pregnancy allows time for the patient to learn more about her options and make an informed decision. Immediate postpartum placement of long-acting reversible contraception (LARC), including postplacental IUD placement or Nexplanon, reduces the risk of short-interval unintended pregnancy. Prenatal discussion of this option should be included in contraceptive counseling.

In addition, in the State of Michigan, patients with Medicaid desiring permanent sterilization (eg, tubal ligation) are required to have a signed consent at least 30 days in advance of the procedure.

Choosing a newborn health care provider. To facilitate appropriate follow-up of infants, the identification of a newborn care provider should be made *prior to 36 weeks' gestation.*

COPY

Delivery Planning

Timing of Delivery

Planned delivery of uncomplicated pregnancies (either by induction of labor or cesarean delivery) should be *avoided before 39 weeks' gestation.* For women with uncomplicated pregnancies, induction of labor should be offered at *41 weeks' gestation.* However, it is reasonable to offer induction even at 39 weeks' gestation; an NIH study showed lower rates of cesarean delivery and hypertensive disorders of pregnancy in women electively induced at 39 weeks. Induction of labor should be strongly recommended to women by *42 weeks' gestation*. Comparing induction of labor at 41 versus 42 weeks, 41-week induction results in:

- Less meconium-stained amniotic fluid (RR 0.50)
- No difference in neonatal intensive care admissions
- No change in cesarean delivery rates (slightly lower in 41 week inductions)
- No difference in operative vaginal delivery
- Less fetal demise, but absolute risk is small (NNT = 410)

Gestational Age Determination
The gestational age-based estimated date of delivery (EDD) should be established *prior to 20 weeks'* gestational age and reviewed prior to planning any intervention.

- In vitro fertilization is expected to be accurate to ± 1 day.
- Ovulation induction, artificial insemination, a single intercourse record, ovulation predictor assay, or basal body temperature measurement are typically accurate to ± 3 days.
- Last menstrual period (LMP) dating is dependent on accurate recollection of a definite normal LMP and regular 28-day cycles when not taking hormonal contraceptives.
- Ultrasound performed by a trained sonographer is considered to be consistent with LMP dating if there is agreement to within the timeframe described in Table 6. If dates are not consistent, refer to results of the initial ultrasound examination.

Table 6. Gestational Age Determination by Ultrasound

Gestational Age (weeks)	Expected Variation in Sonographic Measurement
6-10	± 3 days by crown-rump length
10-14	± 5 days by crown-rump length
14-21	± 7 days by the average of multiple biometric parameters
21-28	± 14 days by the average of multiple biometric parameters
> 28 weeks	± 21 days by the average of multiple biometric parameters

For patients with sonographic dating established at or beyond 24 weeks, a second ultrasound examination is suggested 3-6 weeks later to evaluate for appropriate growth.

COPY

Membrane Sweeping

Membrane sweeping may be offered to women at *every visit beginning at 38 weeks' gestation*. Membrane sweeping decreases need for postdates induction of labor (NNT = 8). However, patients should be counseled on the potential for pain, cramping, and spotting after membrane sweeping.

Repeat cesarean delivery and vaginal birth after cesarean delivery (VBAC). A trial of labor after cesarean delivery (TOLAC) should be offered to women who have both:

- 2 or fewer prior cesarean deliveries, AND
- Prior low transverse incision, as documented in an operative note. In cases where this documentation is not available, the patient should have NO known history of a delivery where a classical cesarean incision would typically have been used. A classical cesarean incision is made vertically and is usually used only in complicated situations such as rapid or emergency delivery, placenta previa, or when the fetus is very preterm or has certain congenital anomalies.

Potential for successful VBAC can be assessed prenatally using the validated NIH VBAC calculator at: https://mfmu.bsc.gwu.edu
Compared to scheduled repeat cesarean delivery, **benefits** of a successful TOLAC include:

- Faster recovery after birth
- Shorter hospital stay
- Decreased risk for infection after delivery
- Decreased risk for blood transfusion
- Decreased risk for surgical complications
- Decreased risk for neonatal respiratory complications
- Quicker return to normal activities
- Greater chance of having vaginal birth in later pregnancies
- Decreased risk for abnormal placentation in future pregnancies

The **risks** of TOLAC increase if unsuccessful, and include

- Uterine rupture (occurs at a rate of 0.5-1%)
- Blood loss requiring transfusion
- Damage to the uterus requiring hysterectomy
- Bladder injury
- Infection
- Increased risk for hypoxic ischemic encephalopathy in the newborn

Counsel eligible patients on risks and benefits of TOLAC at the *initial visit, at 28 weeks' gestation, and once again near term.* If a patient chooses to pursue a trial of labor, a signed informed consent document that delineates the risks and benefits is recommended. Induction of labor increases the risk of uterine rupture to approximately 1.4%. The likelihood of successful VBAC should occur again in the setting of induction using the VBAC calculator. At that time a discussion of the likelihood of success in conjunction with the increased risk of uterine rupture should occur prior to beginning the induction.

COPY

Postpartum Assessment

Recommended postpartum follow up is a contact at *10-14 days after delivery* and an office visit *4 weeks' postpartum*. Timing of the office visit has traditionally been between 6-8 weeks, but patients may benefit from earlier surveillance for postpartum depression, breastfeeding issues and/or contraception initiation.

The following should be included in the postpartum visit:

- Pelvic and breast examinations as needed
- Cervical cytology should be completed at six to eight weeks' postpartum if indicated by cervical cancer screening guidelines.
- Screening for postpartum depression
- Screening for intimate partner violence
- Patients with pregnancies complicated by gestational diabetes should be tested for diabetes using a two-hour 75 g oral glucose tolerance test between *4-12 weeks' postpartum.*

- Patients with pregnancies complicated by hypertensive disorders should be counseled on long-term cardiovascular risks.

The visit should also include education about contraception, infant feeding, sexual activity, weight, and exercise.

Indications for Referral to High-Risk Provider

In general, prenatal care can be provided by appropriately trained and knowledgeable health care providers. However, certain situations require consultation and management by a physician trained in the care of high-risk obstetrical patients. Any aspect of prenatal care which is outside the scope of the provider's usual practice is an indication for referral. Table 3 lists common conditions that warrant consideration of high-risk provider consultation.

Cultural Sensitivity

Understanding the cultural context of particular patient's health-related behavior can improve patient communication and care. Health care providers can minimize situations that strain provider-patient relationships by increasing their understanding and awareness of the cultures they serve and by being open minded and educating themselves regarding those that they do not know.

Provide patient-centered care and honor cultural differences as long this does not result in discrimination against staff and providers.

Adult patients have the right to make decisions about their own health care. A model of shared decision making and support for patient autonomy should guide all patient-provider interactions.

COPY

Related National Guidelines

This guideline generally conforms to:

VA/DoD practice Guideline for Pregnancy Management (2009, 2018)

Guidelines for perinatal care. AAP/ACOG (2017)

Performance Measures

National programs with related clinical performance measures include the following:

- Centers for Medicare & Medicaid Services (CMS)
- National Committee for Quality Assurance: Healthcare Effectiveness Data and Information Set (HEDIS)
  Regional programs that have clinical performance measures of prenatal care include the following.
- Blue Cross Blue Shield of Michigan (BCBSM)
- Blue Care Network [HMO]: clinical performance measures (BCN)

These programs have clinical performance measures for prenatal care and general preventive care addressed in this guideline. While specific measurement details vary (eg, method of data collection,

population inclusions and exclusions), the general measures are summarized below.

Screening measures specific to prenatal care are:

Timeliness of Prenatal Care: The percentage of deliveries that received a prenatal care visit as a member of the organization in the first trimester, on the enrollment start date or within 42 days of enrollment in the organization. (HEDIS)

Postpartum Care: The percentage of deliveries that had a postpartum visit on or between 21 and 56 days after delivery. (HEDIS)

HBsAg testing in Pregnancy: All female patients aged 12 and older who had a live birth or delivery during the measurement period who were tested for hepatitis B surface antigen (HBsAg) during pregnancy within 280 days prior to delivery (CMS)

Prenatal/Postpartum Depression Screening: The proportion of live deliveries between 2-14 months prior to the measurement end date for patients ≥ 12 years of age, who have also had depression screenings (PHQ2, PHQ9, or EPDS) performed during each of these three time periods: 1) between 1 year prior to the Estimated Delivery Date (EDD) through the second trimester; 2) between the beginning of the third trimester through the delivery date; and 3) between delivery date and 56 days postpartum.

Screening measures that apply to all patients are:

COPY

Adult Cervical Cancer Screening: Percentage of women who either (1) had cervical cytology performed every 3 years (ages 21-64), or (2) had cervical cytology/human papillomavirus (HPV) co-testing performed every 5 years (ages 30-64). (HEDIS, BCBSM)

Blood Pressure Screening: The percentage of patients 18–85 years of age who had a diagnosis of hypertension (HTN) and whose BP was adequately controlled during the measurement year. BP control is defined as: 1) BP < 140/90 mm/Hg for patients 18 - 59 years of age; 2) BP < 140/90 mmHg for patients 60 - 85 years of age with diabetes; and 3) BP < 150/90 mm/Hg for patients 60 - 85 years of age without diabetes. (HEDIS, BCBSM, BCN, CMS)

BMI Screening: The percentage of members 18–74 years of age who had an outpatient visit and whose body mass index (BMI) was documented during the measurement year or the year prior to the measurement year. (HEDIS, BCBSM, BCN)

Chlamydia Screening: The percentage of women 16–24 years of age who were identified as sexually active and who had at least one test for chlamydia during the measurement year. (HEDIS)

Depression Screening: Percentage of patients ages 12 and older screened for clinical depression using an age appropriate standardized tool AND follow-up plan documented (CMS)

Influenza Vaccination: Percentage of patients aged 6 months and older seen for a visit between October 1 and March 31 of the one-year measurement period who received an influenza immunization OR who reported previous receipt of an influenza immunization. (CMS)

Tobacco Use: Percentage of patients aged 18 years and older who were screened for tobacco use one or more times within 24 months AND who received cessation counseling intervention if identified as a

tobacco user (HEDIS, CMS)

Literature Search

For this update the initial evidence base was the literature search performed to develop the 2006 version of this guideline. The team accepted the literature search performed to produce the Veterans Administration/Department of Defense (VA/DoD) Practice Guideline for Pregnancy Management (2009, 2018; see references). That search included literature through December 2007. A Medline search for literature published since that time was performed. The search was conducted prospectively using the major key words of pregnancy (prenatal care); guidelines, controlled trials, cohort studies; published from 1/1/08 through 1/31/12, women (adolescent, adult), English language. Specific searches were performed for: genetic screening & counseling (hemoglobinopathies, cystic fibrosis, Ashkenazi Jews), nutrition counseling (folic acid, calcium supplementation, diet/foods), other counseling (weight gain in pregnancy, exercise, contraception counseling), laboratory studies (rubella titer, hemoglobin/hematocrit, hepatitis B surface antigen, HIV, Rh factor blood type, urine culture or urinalysis, screening for sexually transmitted disease, pap smear, hypothyroidism, TB testing), comorbid conditions (obesity, depression, domestic violence, recurrent preterm birth, herpes simples management), prenatal visits (frequency, urine dipstick, fetal growth assessment, fetal imaging/ultrasound, gestational age determination, screening for aneuploidy, screening for neural tube defects, screening for diabetes/ gestational diabetes, anemia, preeclampsia, gestational hypertension, fetal movement counts, group B streptococcus, breech, membrane sweeping, identification of a pediatrician), delivery (timing, repeat cesarean delivery and vaginal birth after cesarean delivery, elective primary cesarean delivery), breast feeding, indications for referral to high-risk care, cultural sensitivity.

The searches were supplemented with recent clinical trials known to expert members of the panel. The search was single cycle. Conclusions were based on prospective randomized clinical trials if available, to the exclusion of other data. If RTC were not available, observational studies were admitted to consideration. If no such data were available, expert opinion was used to estimate effect size.

Disclosures

No member of the Prenatal Care Guideline Team has relationships with commercial companies whose products are discussed in this guideline. The members of the team are listed on the front page of this guideline.

Review and Endorsement

Drafts of this guideline were reviewed in clinical conferences and by distribution for comment within departments and divisions of the University of Michigan Medical School to which the content is most relevant: Family Medicine, Obstetrics/Gynecology, and Pediatrics. The guideline was approved by the Perinatal Joint Practice Committee and the Executive Committee of the University of Michigan C. S. Mott Children's Hospital and Von Voigtlander Women's Hospital. The final version was endorsed by the Clinical Practice Committee of the University of Michigan Faculty Group Practice and the Executive Committee for Clinical Affairs of the University of Michigan Hospitals and Health Centers.

COPY

Acknowledgments

The following individuals are acknowledged for their contributions to previous versions of this guideline.

1997:Patricia Crane, MSN, Nursing; Jennifer Hoock, MD, Family Medicine; Dolores Mendelow, MD, Pediatrics; Connie Standiford, MD, General Internal Medicine; Christopher Wise, PhD, Clinical Affairs; Mary Ann Zettelmaier, CNS, Nursing.

1999:Robert Hayashi, MD, Obstetrics/Gynecology; Stephen Park, MD, Pediatrics; Robert Schumacher, MD, Pediatrics; and Renee Stiles, PhD, Clinical Affairs.

2006:Lauren B. Zoschnick, MD, Obstetrics/Gynecology; Erin L. Brackbill, MD, Pediatrics; Lee A. Green, MD, Family Medicine; R. Van Harrison, PhD, Medical Education; Robert E. Schumacher, MD, Pediatrics.

Annotated References

The Pregnancy Management Working Group. VA/DoD Practice Guideline for Pregnancy Management. Washington DC: US Department of Veterans Affairs and Department of Defense, 2009.

This document summarizes evidence and recommendations for the management of uncomplicated pregnancy.

This document was updated in 2017 and was substantially truncated to only 22 bullet points of recommendation. The 2009 version remains a comprehensive resource.

ACOG Committee opinion no. 549: Obesity in pregnancy. Obstetrics & Gynecology, 2013; 121(1):213-217.

Recommendations regarding obesity in pregnancy.

American Academy of Pediatrics / American College of Obstetricians and Gynecologists (editors). Guidelines for Perinatal Care, Eighth Edition. Washington, DC: American College of Obstetricians and Gynecologists, 2017.

Comprehensive national guidelines for perinatal care from ACOG and the AAP.

American College of Obstetricians and Gynecologists. Hypertension in pregnancy: executive summary. Obstet Gynecol. 2013 Nov;122(5):1122-31.

Comprehensive guideline on the management of hypertensive disorders in pregnancy from ACOG.

Caring for our future: The content of prenatal care. A report of the Public Health Service expert panel on the content of prenatal care. Department of Health and Human Services, Washington, D.C. 1989.

A report on effective and efficient approaches for prenatal care, developed by the Public Health Service expert panel.

Committee opinion No. 561: Nonmedically indicated early-term deliveries. Obstetrics & Gynecology 2013; 121(4):911-5.

ACOG statement addressing scheduled deliveries prior to 39 weeks.

COPY

Dowswell T, Carroli G, Duley L, et al. Alternative versus standard packages of antenatal care for low-risk pregnancy. Cochrane Database of Systematic Reviews 2015, Issue 7. Art. No.: CD000934. DOI: 10.1002/14651858.CD000934.pub3.

Grobman WA, Rice MM, Reddy UM, et al; Eunice Kennedy Shriver National Institute of Child Health and Human Development Maternal–Fetal Medicine Units Network. Labor Induction versus Expectant Management in Low-Risk Nulliparous Women. N Engl J Med. 2018 Aug 9;379(6):513-523. doi: 10.1056/NEJMoa1800566.

Hollier LM, Wendel GD. Third trimester antiviral prophylaxis for preventing maternal genital herpes simplex virus (HSV) recurrences and neonatal infection. Cochrane Database of Systematic reviews, 2008; Jan 23(1):CD004946.

Cochrane Database review of antiviral prophylaxis for patients with herpes simplex virus in pregnancy.

Kim DK, Riley LE, Hunter P. Advisory Committee on Immunization Practices Recommended Immunization Schedule for Adults Aged 19 Years or Older — United States, 2018. MMWR Morb Mortal Wkly Rep 2018;67:158–160. DOI:http://dx.doi.org/10.15585/mmwr.mm6705e3.

Kirkham C, Harris S, Grzybowski S. Evidence-based prenatal care: Part I. General prenatal care and counseling issues. Am Fam Physician. 2005 Apr 1;71(7):1307-16.

Evidence-based review of many aspects of prenatal care.

Koopmans CM, Bijlenga D, Groen H, et al. Induction of labour versus expectant monitoring for gestational hypertension or mild pre-eclampsia after 36 weeks' gestation (HYPITAT): a multicentre, open-label randomised controlled trial. Lancet. 2009; 374(9694):979-88.

Randomized controlled trial of delivery of patients with mild gestational hypertension at term.

Landon MB, Gabbe SG. Gestational diabetes mellitus. Obstet Gynecol. 2011 Dec;118(6):1379-93.

Evidence-based review of the diagnosis and treatment of gestational diabetes.

Schwenk TL, Terrell LB, Harrison RV, Tremper AL, Valenstein MA. Depression [2011 update]. Ann Arbor, Michigan: University of Michigan Health System, 2012. (Available at: www.med.umich.edu/1info/fhp/practiceguides/ccg.html)

Serlin DC, Clay MA, Harrison RV, Thomas LA. Tobacco Treatment [2012 update]. Ann Arbor, Michigan: University of Michigan Health System, 2012. (Available at: www.med.umich.edu/1info/fhp/practiceguides/ccg.html)

Society for Maternal-Fetal Medicine Publications Committee with assistance of Vincenzo Berghella. Progesterone and preterm birth prevention: translating clinical trials data into clinical practice. Am J Obstet Gynecol. 2012 May;206(5):376-86.

SMFM statement reviewing progesterone and its role in the prevention of preterm birth.

Standiford CJ, Vijan S, Choe HM, Harrison RV, Richardson CR, Wyckoff JA. Management of Type 2 Diabetes Mellitus [2012 update]. Ann Arbor, Michigan: University of Michigan Health System, 2012.

COPY

(Available at: www.med.umich.edu/1info/fhp/practiceguides/ccg.html)

Standiford CJ, George-Nwogu UD, Vijan S, Rew KT, Harrison VH. Cancer Screening [2012 update]. Ann Arbor, Michigan: University of Michigan Health System, 2012. (Available at: www.med.umich.edu/1info/fhp/practiceguides/ccg.html)

Internet Citation: *Recommendation Summary*. US Preventive Services Task Force. September 2014.

http://www.uspreventiveservicestaskforce.org/Page/Topic/recommendation-summary/low-dose-aspirin-use-for-the-prevention-of-morbidity-and-mortality-from-preeclampsia-preventive-medication

Wald NJ, Law MR, Morris JK, Wald DS. Quantifying the effect of folic acid. Lancet, 2001; 358: 2069-73.

Review of studies of folic acid supplementation on risk of neural tube defects to determine effective dosing.

Carrier screening for genetic conditions. Committee Opinion No. 691. American College of Obstetricians and Gynecologists. Obstet Gynecol, 2017;129:e41–55.

Initial Release

December 2013

Interim/Minor Revision

October 2015

January 2019

May 2021

COPY

Literature search service: Taubman Health Sciences Library

© Regents of the University of Michigan

Approval Signatures

Step Description	Approver	Date
PolicyStat Administrator Review	Natalie Plata: Clinical Policy Manager	06/2022
PolicyStat Administrator Review	Olivia Curl: Admin Coord/ Project Coord	06/2022

initial owner review	Joanne Bailey: Collegiate Lecturer, Nurse Midwife Director and Le	06/2022

COPY
