# Supplementary material for: Analysis of disparities in the utilization of virtual prenatal visits in pregnancy
Source: AJOG Glob Rep. 2022 Dec 5;3(1):100142. doi: 10.1016/j.xagr.2022.100142 (PMC9823200; doi:10.1016/j.xagr.2022.100142)
Supplement: Supplementary file 2 [file mmc2.docx]

**Appendix B. Demographic comparisons between included patients who were eligible for virtual care and excluded patients who were not eligible for virtual care**

| Group Patient Characteristics | Included (n=3880)^a^ | Excluded (n=648)^a^ | P value^b^ |
| --- | --- | --- | --- |
| Age, years |  |  | **<0.001** |
| ≤20 | 62 (1.6) | 18 (2.8) |  |
| 21-29 | 1204 (31.0) | 189 (29.2) |  |
| 30-34 | 1547 (39.9) | 221 (34.1) |  |
| 35-39 | 909 (23.4) | 169 (26.1) |  |
| ≥40 | 158 (4.1) | 51 (7.9) |  |
| Race |  |  | **0.048** |
| White or Caucasian | 2808 (72.4) | 478 (73.8) |  |
| Black or African American | 462 (11.9) | 90 (13.9) |  |
| Other^c^ | 610 (15.7) | 80 (12.4) |  |
| Ethnicity |  |  | 0.657 |
| Hispanic | 209 (5.4) | 40 (6.2) |  |
| Non-Hispanic | 3641 (93.8) | N/A^d^ |  |
| Other^e^ | 30 (0.8) | 602 (92.9) |  |
| Primary Language |  |  | 0.785 |
| English | 3703 (95.4) | 620 (95.7) |  |
| Non-English | 177 (4.6) | 28 (4.3) |  |
| Insurance |  |  | <0.001 |
| Commercial | 2522 (65) | 376 (58.0) |  |
| Medicaid | 741 (19.1) | 201 (31.0) |  |
| Uninsured | 603 (15.5) | 66 (10.2) |  |
| Unknown | 14 (0.4) | N/A^d^ |  |
| Parity |  |  | **0.006** |
| Nulliparous | 2287 (58.9) | 229 (35.3) |  |
| Multiparous | 1593 (41.4) | 419 (64.7) |  |
| Marital Status |  |  | **0.002** |
| Married/significant other | 1664 (42.9) | 257 (39.7) |  |
| Single/separated/other | 1073 (27.7) | 156 (24.1) |  |
| Unknown | 1143 (29.5) | 235 (36.3) |  |

^a^Data presented as n (%)

^b^P value for bivariate analysis; calculated using Chi-squared test; bold indicates significance at P<0.05

^c^Institutional standard selections for race (American Indian or Alaskan Native, Asian, Native Hawaiian or Other Pacific Islander, multiracial, self-described other, unknown, choose not to disclose)

^d^N/A due to n<11

^e^Institutional standard selections for ethnicity (self-described other, unknown, choose not to disclose)
